# Genetic testing and exchangeable copper can improve the accuracy of Wilson disease diagnoses

**DOI:** 10.1097/HC9.0000000000001045

**Published:** 2026-09-18

**Authors:** Marina Berenguer, Mercè Torra, Carmen Espinós, Rocío Andreu Escrivá, Anna Pocurull, Ângela Carvalho-Gomes, Anna Miralpeix, Gustavo Rojas Echarry, Sarai Romero-Moreno, Clara Sanchez, Eva Silgo, Christian Palacios-Martínez, Celia Badenas, Judith Perez-Rojas, Cristina Aguado Codina, Zoe Mariño

**Affiliations:** 1 Hepatology Unit, Department of Gastroenterology, La Fe University Hospital, Valencia, Spain; 2 Hepatology, Hepatobiliopancreatic Surgery and Transplant Group, La Fe Health Research Institute (IIS La Fe), Valencia, Spain; 3 Centro de Investigacion Biomedica en Red de Enfermedades Hepáticas y Digestivas (CIBEREHD), Instituto de Salud Carlos III (ISCIII), Madrid, Spain; 4 Department of Medicine, University of Valencia, Valencia, Spain; 5 European Reference Network on Rare Liver Disorders (ERN RARE-LIVER), Hamburg, Germany; 6 Biochemistry and Molecular Genetics Unit, Hospital Clínic Barcelona, IDIBAPS, Barcelona, Spain; 7 Centro de Investigación Biomédica en Red de Enfermedades Raras (CIBERER), Instituto de Salud Carlos III (ISCIII), Madrid, Spain; 8 Department of Genetics, Universitat de València, Valencia, Spain; 9 Biomedical Research Institute INCLIVA, Valencia, Spain; 10 Clinical Laboratory Unit, La Fe University Hospital, Valencia, Spain; 11 Liver Unit, Hospital Clínic, IDIBAPS, Universitat de Barcelona, Barcelona, Spain; 12 Department of Pathology, La Fe University Hospital, Valencia, Spain; 13 Laboratory of Rare Neurodegenerative Diseases, Centro de Investigación Príncipe Felipe, Valencia, Spain

**Keywords:** ATP7B, ceruloplasmin, cirrhosis, genetics, liver disease, neurology

## Abstract

**Figure:**
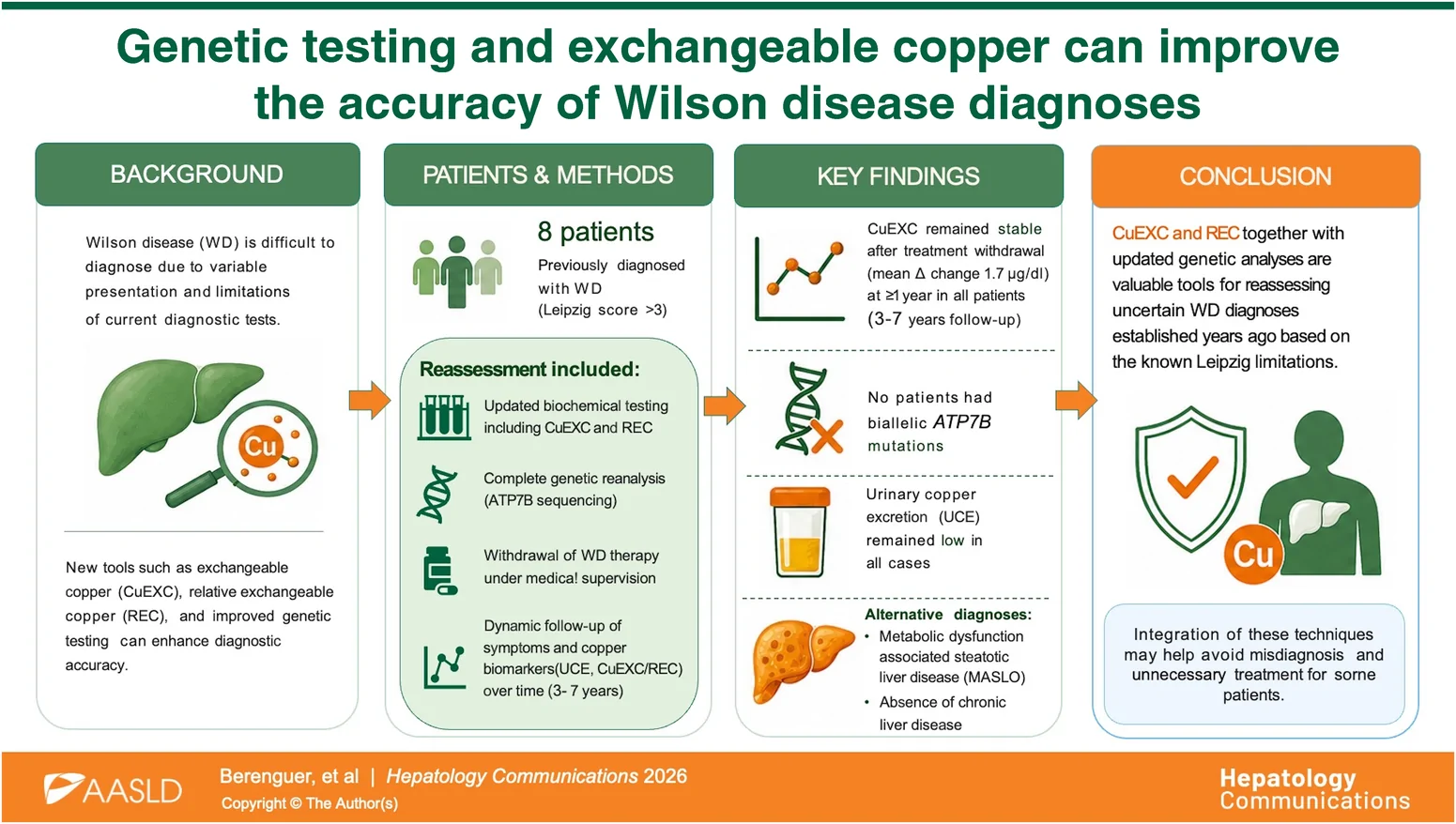

Wilson disease (WD) is an inherited disorder of copper metabolism caused by pathogenic ATP7B variants, leading to impaired biliary copper excretion and progressive hepatic and neuropsychiatric manifestations.^1^ Although the Leipzig score remains a useful diagnostic framework, WD diagnosis may be challenging in patients with incomplete phenotypes, borderline biochemical findings, or competing liver diseases.^1^ This is particularly relevant for patients diagnosed decades ago, when comprehensive ATP7B sequencing, copy-number analysis, and direct assessment of exchangeable copper were not routinely available.^2^ Consequently, reliance on isolated biochemical abnormalities may have led to overdiagnosis and unnecessary lifelong treatment.

Exchangeable copper (CuEXC) and relative exchangeable copper (REC; CuEXC/total serum copper ratio) are useful biomarkers for WD diagnosis.^3^,^4^,^5^,^6^ REC has shown high accuracy in untreated patients, with diagnostic thresholds ranging from 15% to 18.5%.^1^,^3^,^4^,^5^,^6^ However, interpretation in treated individuals remains uncertain because long-term therapy may alter copper fractions and REC kinetics after treatment withdrawal are poorly defined.^1^,^3^,^4^,^5^,^6^,^7^ We describe 8 patients with a historical diagnosis of WD in whom the diagnosis was reconsidered using updated clinical, biochemical, and genetic assessment.

Patients followed at 2 academic liver units were retrospectively reviewed. All had been diagnosed with WD >10 years earlier, were asymptomatic at diagnosis, and had been investigated because of persistently elevated liver enzymes. Inclusion criteria were: historical Leipzig score ≥4, incomplete biochemical response despite reported adherence, absence of confirmed biallelic ATP7B pathogenic variants in historical records, and suspicion of an alternative liver disease. Reassessment included CuEXC and REC when available. Genetic re-analysis comprised updated ATP7B sequencing and MLPA, including splice-site variants at exon–intron boundaries; variants were classified according to ACMG/AMP criteria.^2^,^8^ Based on the combined reassessment, anti-copper therapy was discontinued under supervision. Follow-up reflected the retrospective nature of the cohort and was not uniform. In recent cases, CuEXC and 24-hour urinary copper (UCE) were monitored every 3–6 months.

All patients were male, and none had Kayser–Fleischer rings at diagnosis (Supplemental Table S1, https://links.lww.com/HC9/C484). Age at diagnosis ranged from 17 to 58 years. Median duration of WD-therapy before discontinuation was 19.5 years. Before withdrawal, median ceruloplasmin, UCE, and CuEXC were 0.23 g/L, 98 µg/24 h, and 4.4 µg/dL, respectively.

Follow-up ranged from 35 to 95 months (Supplemental Tables S1 and S2, https://links.lww.com/HC9/C484). No patient developed hepatic decompensation, progressive biochemical deterioration, or clinical features suggestive of active WD. Copper biomarkers remained consistently outside the expected range for true WD. REC values were low and stable (3.0%–8.6%), without progressive increases, while CuEXC remained within or below the range reported for non-WD individuals (Figure 1).^9^ UCE decreased after chelator withdrawal, consistent with loss of drug-induced cupriuresis, and remained below 100 µg/24 h in all patients.

**FIGURE 1:**
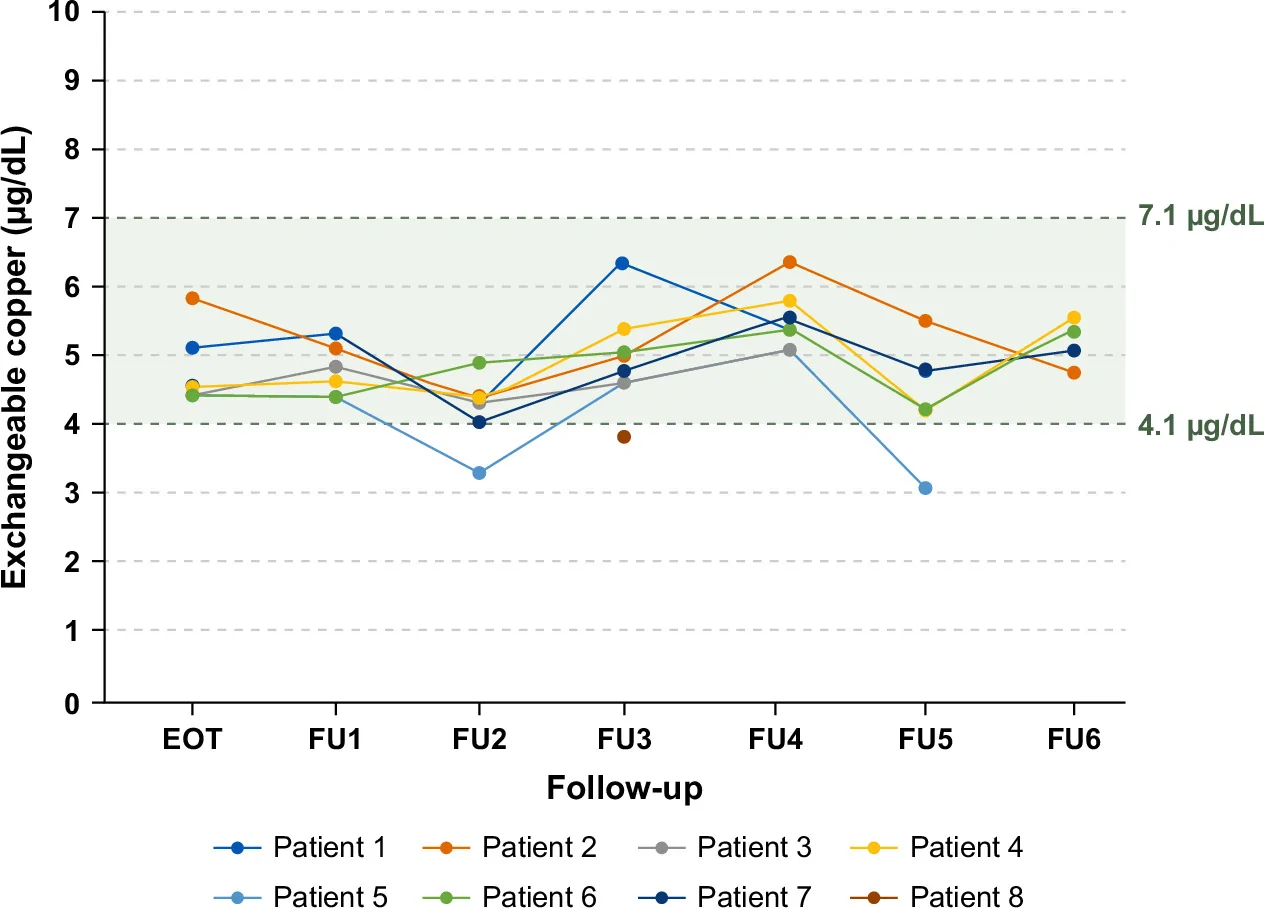
Post-treatment variations in exchangeable copper (CuEXC). *Y*-axis: CuEXC (µg/dL). *X*-axis: time points during follow-up. Abbreviations: CuEXC, exchangeable copper; EOT, end-of-treatment; FU, follow-up.

Updated genetic testing did not identify pathogenic biallelic ATP7B variants in any case. Five patients had no detectable ATP7B variants, whereas 3 carried a single heterozygous variant or a variant of uncertain significance (VUS), insufficient to support a molecular diagnosis of WD in the absence of disturbed copper metabolism. In 2 patients, repeat biopsy before discontinuation showed hepatic copper concentrations below the diagnostic threshold. Alternative diagnoses were identified in most cases (MASH in 5, MetALD in 1, no chronic liver disease in 2). Overall, 6 patients had metabolic risk factors or histological/imaging features compatible with MASH. Lifestyle intervention, weight loss, and risk-factor control were associated with improvement of liver enzymes.

These cases illustrate that a historical WD diagnosis should be reconsidered when the clinical course is atypical, treatment response is incomplete, and contemporary genetic testing fails to confirm biallelic ATP7B disease. Persistently low REC values, together with stable CuEXC, low UCE after treatment withdrawal, and identification of a more plausible alternative liver disease, may support diagnostic reassessment.^4^,^6^,^7^,^8^,^10^ The absence of biallelic pathogenic ATP7B variants further strengthens this interpretation.^2^,^10^

Nevertheless, genetic testing is not infallible. More than 900 ATP7B pathogenic variants have been described; interpretation may be challenging because of technical limitations, VUS, or undetected pathogenic variants. Furthermore, heterozygous carriers may exhibit mild abnormalities in copper metabolism that can contribute to false-positive Leipzig scores if not interpreted carefully.^1^,^2^,^10^

Treatment withdrawal should not be based on REC alone. Some true WD patients, particularly those treated for many years or diagnosed presymptomatically, may exhibit REC values below conventional thresholds.^6^,^7^ Therefore, stopping anti-copper therapy should only be considered after expert multidisciplinary review and long-term monitoring.

In conclusion, integration of CuEXC, REC, and updated ATP7B testing may improve reassessment of uncertain historical WD diagnoses. In selected patients with low diagnostic probability after comprehensive re-evaluation, supervised treatment withdrawal may be feasible, avoiding unnecessary lifelong therapy and redirecting care toward the correct underlying liver disease.

## Supplementary Material
