# Supplementary material for: Genetic testing and exchangeable copper can improve the accuracy of Wilson disease diagnoses
Source: Hepatol Commun. 2026 Sep 18;10(10):e1045. doi: 10.1097/HC9.0000000000001045 (PMC13592991; doi:10.1097/HC9.0000000000001045)
Supplement: Supplementary file 1 [file hc9-10-e1045-s001.docx]

**Suppl Table 1. Baseline features and longitudinal clinical and histological follow up following treatment discontinuation**

**Legend:** AHT= arterial hypertension; BMI= body mass index; CuEXC= Exchangeable copper (normal range: 4-7 ug/dl); D/C= discontinuation; DL: dyslipidemia; DPA= D-Penicillamine;; M= male; MASLD= metabolic associated steatotic liver disease; MetALD=metabolic dysfunction-associated steatotic liver disease and simultaneous moderate alcohol consumption; MASH=metabolic dysfunction-associated steatohepatitis; REC=relative exchangeable copper {ratio of CuEXC/ total copper (or REC, expressed in %)}; UCE= urinary copper excretion

**Suppl Table 2. Longitudinal analytical liver and copper metabolism related data at end of therapy and following treatment discontinuation**

**Legend:** ALT= alanine aminotransferase; AST= aspartate aminotransferase; Bil: bilirubin; CuEXC= Exchangeable copper (normal range: 4-7 ug/dl); DPA= D-Penicillamine; PT= prothrombin time; REC=relative exchangeable copper {ratio of CuEXC/ total copper (or REC, expressed in %)}; UCE= urinary copper excretion

**Suppl Table 1.**

|  | **Age at diagnosis** | **Sex (M/F)** | **Comorbidities/ Family history** | **KF ring** | **Leipzig score without genetic studies** | **Genetic mutation** | **Treatment at diagnosis** | **Maintenance therapy (up to D/C)** | **Total time on therapy (yrs)** | **Post D/C outcome** | **Comorbidities at time of D/C (obesity/DM/ alcohol)** | **Final diagnosis** | **Biopsy at diagnosis** | | **Biopsy at/after D/C (if available)** | | |
| --- | --- | --- | --- | --- | --- | --- | --- | --- | --- | --- | --- | --- | --- | --- | --- | --- | --- |
|  |  |  |  |  |  |  |  |  |  |  |  |  | **Intra-hepatic Cu (microg/g)** | **Description** | **Intra-hepatic Cu (microg/g)** | **Description** | |
| 1 | 23 | M | Obesity | No | 4 | No | Zinc (compounded formulation) | Zinc (acetate) | 17 | Clinical stability | Grade 3 obesity (BMI 41.95 Kg/m^2^) | MASH | ND | Liver parenchyma with fibrous expansion of portal tracts, without bridging or signs of cirrhosis. Severe macrovesicular steatosis with minimal lobular inflammation | ND | ND | |
| 2 | 40 | M | DL | No | 4 | p.G869R in heterozygosis | DPA | Zinc (sulphfate) | 19 | Clinical stability | No | No liver disease | 310 | Normal Biopsy | ND | ND | |
| 3 | 18 | M | Obesity, Celiac disease | No | 4 | No mutations | DPA | DPA | 9 | Clinical stability | Grade 1 obesity (BMI 31.4 Kg/m^2^) | MASH | 275 | Discrete chronic portal lymphocytic infiltrate. Mixed lobular inflammatory aggregates | ND | ND | |
| 4 | 17 | M | Obesity, DL | No | 4 | p.H1069Q in heterozygosis | DPA | DPA | 22 | Clinical stability | Overweight (BMI 27-29 Kg/m^2^), alcohol | MetALD | 187 | NA | 54,00 | | Mild steatosis, No steatohepatitis. F 0/4. |
| 5 | 18 | M | Obesity | No | 4 | No mutations | DPA | Zinc (acetate) | 23 | Clinical stability + progressive improvement in liver enzymes with weight loss | Overweight (BMI 29 Kg/m2) | MASH | 600 | Mild steatohepatitis | On therapy 18.5 microg/g | On therapy Grade 3 macro vesicular steatosis | |
| 6 | 35 | M | Obesity, DL and AHT | No | 4 | No mutations | DPA (D/C to nephrotic syndrome) followed by trientine 2HCL | Zinc (acetate) | 22 | Clinical stability; no major transaminase changes concomitant with suboptimal response to MS therapy | Overweight (BMI 29 Kg/m2), suboptimal control AHT & DL well | MASH | 781 | Mild steatohepatitis | ND | ND | |
| 7 | 36 | M | DL | No | 4 | No mutations | Zinc (sulphateulfate) + DPA | Zinc (sulphate) + DPA | 20 | Clinical stability and normal liver enzymes | No | No liver disease | 1312 | Normal biopsy | ND | ND | |
| 8 | 58 | M | Obesity | No | 4 | No mutations | Zinc (acetate) | DPA | 1.8 | Clinical stability with progressive normalization of liver enzymes | Overweight (BMI 28 Kg/m2) | MASH | 1768 | Grade 4 macrovesicular steatosis. No steatohepatitis  F1/4 | On therapy: 18 microg/g | Grade 3 macrovesicular steatosis, mild steatohepatitis, F 3/4 | |

*At diagnosis: p.K1437K-(ATP7B VUS, in heterozygosis;

AHT=arterial hypertension; BMI=Body mass index; D/C= discontinuation; DL=dyslipidaemia; DM=Diabetes Mellitus; DPA= D-Penicillamine; EOT=end-of-treatment; ETG=ethyl glucuronide; F=fibrosis; KF= Kayser Fleisher; MASH=metabolic-associated steatohepatitis; MS=metabolic syndrome; NA=not available; ND=not done

**Suppl Table 2.**

|  |  | **Treatment** | **Date blood tests or months from end of treatment** | Ceruloplasmin (g/L) | 24hr urine Cu (μg/24h) | CuExC (μg/dL) | REC (%) | AST/ALT (U/L) | Total Bil (mg/dL) | PT (%) | Platelet count (10^9/L) |
| --- | --- | --- | --- | --- | --- | --- | --- | --- | --- | --- | --- |
| **1** | At diagnosis | Zinc | 2005 | 0.17 | 963 after DPA (yet spontaneous UCE 23) | NA | NA | 48/114 | 0.8 | NA | 245 |
|  | Prior to discontinuation | Zinc | 17 yrs post-diagnosis | 0.18 | 60 | 3.4 | 5 | 21/34 | 0.97 | 100 | 252 |
|  | Date of drug discontinuation | Zinc | 4 months later | 0.19 | 45 | 5 | 6.3 | 23/39 | 0.9 | 100 | 212 |
|  | Months post- discontinuation |  | 3 | 0.22 | 60 | 5 | 6.3 | 26/43 | 0.6 | 100 | 300 |
|  | Months post- discontinuation |  | 6 | 0.20 | 44 | 4.6 | 6.0 | 25/41 | 1.1 | 100 | 240 |
|  | Months post-discontinuation |  | 13 | 0.19 | 32 | 6.4 | 8.0 | 28/47 | 1.0 | 100 | 212 |
|  | Months post-discontinuation |  | 26 | 0.22 | 38 | 5.4 | 5.5 | 14/ 13 | 0.8 | 100 | 290 |
|  | Months post- discontinuation |  | 38 | ND | ND | ND | ND | 15/17 | 1.6 | 100 | 275 |
| **2** | At diagnosis | DPA | 2005 | 0.23 | 115 | NA | NA | 28/47 | 0.5 | 100 | 291 |
|  | Prior to discontinuation | Zinc | 17 yrs post-diagnosis | 0.23 | 40 | 4.4 | 5.1 | 26/42 | ND | ND | 204 |
|  | Date of drug discontinuation | Zinc | 1 month later |  |  |  |  |  |  |  |  |
|  | Months post- discontinuation |  | 3 | 0.23 | 63 | 5.8 | 6.2 | 32/47 | 0.4 | 100 | 209 |
|  | Months post- discontinuation |  | 7 | 0.21 | 44 | 4.6 | 5.2 | 25/33 | 0.6 | 97.2 | 198 |
|  | Months post-discontinuation |  | 14 | 0.24 | 28 | 5.4 | 6.1 | 24/24 | 0.3 | 100 | 178 |
|  | Months post- discontinuation |  | 27 | 0.2 | 76 | 6.4 | 8.2 | 26/31 | 0.5 | 100 | 253 |
|  | Months post- discontinuation |  | 38 | 0.23 | 29 | 5.6 | 6 | 26/34 | 0,6 | 100 | 253 |
| **3** | At diagnosis | DPA | 2012 | 0.21 | 900 after DPA (yet spontaneous UCE 27) | NA | NA | 33/68 | 0.5 | 80 | 293 |
|  | Prior to discontinuation | DPA | 21 yrs post-diagnosis | 0.25 | 322 | NA | NA | 26/50 | ND | 91 | 347 |
|  | Date of drug discontinuation | DPA | 4 months later |  |  |  |  |  |  |  |  |
|  | Months post-discontinuation |  |  | 0.23 | 21 | NA | NA | 25/50 | 0.5 | 96.9 | 366 |
|  | Months post- discontinuation |  | 6 | 0.26 | 24 | NA | NA | 48/41 | 0.4 | 98.4 | 376 |
|  | Months post- discontinuation |  | 13 | 0.27 | 36 | 4.8 | 4.2 | 28/49 | 0.4 | 83.6 | 368 |
|  | Months post-discontinuation |  | 25 | 0.24 | 45 | 5.2 | 4.7 | 20/32 | 0.4 | 100 | 383 |
|  | Months post-discontinuation |  | 45 | 0.24 | 48 | 3 | 3 | 23/38 | 0.5 | 100 | 420 |
| **4** | At diagnosis | DPA | 2000 | 0.18 | 684 after DPA | NA | NA | 36/76 | 0.6 | 100 | 357 |
|  | Prior to discontinuation | DPA | 21 yrs post-diagnosis | 0.30 | 136 | NA | NA | 35/50 | 0.5 | 100 | 336 |
|  | Date of drug discontinuation | DPA | 1 month later |  |  |  |  |  |  |  |  |
|  | Months post- discontinuation |  | 1 | 0.26 | 58 | NA | NA | 33/49 | 0.4 | 100 | 288 |
|  | Months post- discontinuation |  | 5 | 0.25 | 68 | NA | NA | 37/57 | 1.0 | 98.4 | 307 |
|  | Months post-discontinuation |  | 10 | 0.25 | 74 | 5.6 | 5.3 | 41/62 | 0.6 | 95.8 | 311 |
|  | Months post-discontinuation |  | 31 | 0.25 | 24 | 5.8 | 6 | 32/55 | 0.4 | 100 | 340 |
|  | Months post-discontinuation |  | 43 | 0.21 | 51 | 4.2 | 5.3 | 33/58 | 0.5 | 100 | 329 |
|  | Months post-discontinuation |  | 57 | 0.2 | 14 | 5.6 | 6.4 | 31/46 | 0.7 | 100 | 375 |
| **5** | At diagnosis | DPA | 1996 | 0.13 | 200 | NA | NA | 26/70 | 1.18 | 83 | 198 |
|  | Prior to discontinuation | Zinc | 123 yrs post-diagnosis | 0.21 | <10 | NA | NA | 47/118 | 1 | 96 | 271 |
|  | Date of drug discontinuation | Zinc | 11 month later. 2019 |  |  |  |  |  |  |  |  |
|  | Months post- discontinuation |  | 3 | 0.22 | 4.9 | NA | NA | 29/53 | 0.9 | 100 | 297000 |
|  | Months post- discontinuation |  | 22 | 0.23 | 9.4 | 3.2 | 3.3 | 29/55 | 1.2 | 99 | 278 |
|  | Months post-discontinuation |  | 60 | ND | ND | ND | ND | 27/50 | 0.96 | 99 | 268 |
| **6** | At diagnosis | DPA | 2000 | 0.24 | 200 | NA | NA | 48/64 | 1.01 | 85 | 157 |
|  | Prior to discontinuation | Zinc | 22 yrs post-diagnosis | 0.23 | ND | 4.6 | 5.2 | 56/111 | 1 | 100 | 237 |
|  | Date of drug discontinuation | Zinc | 1 month later |  |  |  |  |  |  |  |  |
|  | Months post- discontinuation |  | 8 | 0.22 | 0 | 4.6 | 5.2 | 66/73 | 1 | 94 | 218 |
|  | Months post- discontinuation |  | 21 | 0.26 | 0 | 5 | 5.5 | 47/68 | 0.9 | 100 | 209 |
|  | Months post- discontinuation |  | 35 | 0.26 | 6.5 | 5.4 | 6.2 | 41/62 | 1 | 100 | 210 |
| **7** | At diagnosis | DPA + Zinc | 1997 | 0.15 | 128 | NA | NA | 36/67 | 1.26 | 100 | 279 |
|  | Prior to discontinuation | DPA + Zinc | 24 yrs post-diagnosis | ND | ND | NA | NA | ND/34 | 1.1 | 100 | 232 |
|  | Date of drug discontinuation | DPA + Zinc | 1 month later |  |  |  |  |  |  |  |  |
|  | Months post- discontinuation |  | 45 | 0.21 | 13 | NA | NA | 22/30 | 1.41 | 100 | 260 |
|  | Months post- discontinuation |  | 69 | 0.17 | 0 | 4 | 6.1 | 28/30 | 0.8 | 100 | 259 |
|  | Months post- discontinuation |  | 84 | 0.17 | 0 | 5 | 8.6 | 24/18 | 0.9 | 100 | 239 |
| **8** | At diagnosis | Zinc | 2016 | 0.13 | 61.2 | NA | NA | 40/95 | 0.97 | 100 | 285 |
|  | Prior to discontinuation | DPA | 2 yrs post-diagnosis | 0.13 | <10 | NA | NA | 43/98 | 1.1 | 100 | ND |
|  | Date of drug discontinuation | DPA | 2 months later |  |  |  |  |  |  |  |  |
|  | Months post- discontinuation |  | 5 | 0.16 | 10 | NA | NA | 41/91 | 1.34 | 100 | 316 |
|  | Months post- discontinuation |  | 22 | ND | 0 | NA | NA | 31/53 | 2.40 (Gilbert syndrome) | 100 | 335 |
|  | Months post- discontinuation |  | 60 | 0.17 | 3 | 3.8 | 6 | 37/67 | 1.5 | 100 | 407 |
|  | Months post-discontinuation |  | 73 | 0.19 | 44 | ND | ND | 38/45 | 2.37 | 100 | 356 |
|  | Months post- discontinuation |  | 95 | 20 | 75 | ND | ND | 30/53 | 2.98 | 100 | 372 |
